# Hetero‐Diels–Alder Cycloaddition with RAFT Polymers as Bioconjugation Platform

**DOI:** 10.1002/anie.202005747

**Published:** 2020-09-02

**Authors:** Ana Beloqui, Shivshankar R. Mane, Marcel Langer, Mathias Glassner, Dennis M. Bauer, Ljiljana Fruk, Christopher Barner‐Kowollik, Guillaume Delaittre

**Affiliations:** ^1^ Institute of Biological and Chemical Systems (IBCS) Karlsruhe Institute of Technology (KIT) Hermann-von-Helmholtz-Platz 1 76344 Eggenstein-Leopoldshafen Germany; ^2^ Macromolecular Architectures Institute for Chemical Technology and Polymer Chemistry (ITCP) Karlsruhe Institute of Technology (KIT) Engesserstr. 18 76131 Karlsruhe Germany; ^3^ Department of Applied Chemistry (UPV/EHU) Avda. Manuel de Lardizabal 3 E-20018 Donostia – San Sebastian Spain; ^4^ IKERBASQUE Basque Foundation for Science Maria Diaz de Haro 3 E-48013 Bilbao Spain; ^5^ Center for Functional Nanostructures (CFN) Karlsruhe Institute of Technology (KIT) Wolfgang-Gaede-Straße 1a 76131 Karlsruhe Germany; ^6^ Department of Chemical Engineering and Biotechnology University of Cambridge West Cambridge Site, Philippa Fawcett Drive Cambridge CB3 0AS UK; ^7^ Centre for Materials Science Queensland University of Technology (QUT) 2 George Street Brisbane QLD 4000 Australia; ^8^ School of Chemistry and Physics Queensland University of Technology (QUT) 2 George Street Brisbane QLD 4000 Australia; ^9^ Organic Functional Molecules Organic Chemistry University of Wuppertal Gaußstrasse 20 42119 Wuppertal Germany

**Keywords:** bioconjugation, Diels–Alder cycloaddition, end group, polymer, protein

## Abstract

We introduce the bioconjugation of polymers synthesized by RAFT polymerization, bearing no specific functional end group, by means of hetero‐Diels–Alder cycloaddition through their inherent terminal thiocarbonylthio moiety with a diene‐modified model protein. Quantitative conjugation occurs over the course of a few hours, at ambient temperature and neutral pH, and in the absence of any catalyst. Our technology platform affords thermoresponsive bioconjugates, whose aggregation is solely controlled by the polymer chains.

## Graphical abstract

Already there: Polymers obtained by RAFT polymerization with thiocarbonylthio transfer agents possessing an electron‐deficient carbon–sulfur double bond undergo bioconjugation by hetero‐Diels–Alder cycloaddition, without the need for other reactive end groups. This ligation occurs over a few hours, at ambient temperature and near‐neutral pH, without any catalyst required.

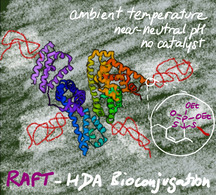

Proteins are essential compounds in modern medicine and biotechnology. However, their physicochemical characteristics brings about critical limitations, particularly in terms of solubility and stability.^[1]^ One of the most important ways of addressing these issues relies on the attachment of synthetic polymer chains, in order to produce so‐called protein‐polymer conjugates (PPCs).[^2^, ^3^, ^4^, ^5^, ^6^] The pioneering and so far most employed polymer for PPCs is polyethylene glycol (PEG). Yet, utilizing other polymers than PEG gives access to a wider range of properties and may elude some shortcomings of PEG,[^7^, ^8^, ^9^, ^10^, ^11^, ^12^] notably its immunogenicity.[^13^, ^14^] In this context, reversible addition‐fragmentation transfer (RAFT) polymerization is one the most powerful synthetic techniques to access macromolecules with defined chain length and (end‐group) functionality.[^15^, ^16^, ^17^] One of the methods to achieve RAFT‐based PPCs involves reacting one end of the synthetic polymer with one or several residues on the protein surface. The reactive end of the RAFT polymer is typically introduced through the reinitiating fragment—the so‐called R group—of a specifically designed chain transfer agent (CTA).^[12]^ The RAFT‐hetero‐Diels–Alder cycloaddition (RAFT‐HDA) emerged about a decade ago as a complementary and highly efficient method for chain‐end conjugation of RAFT polymers without the need for introducing functional R groups.[^18^, ^19^, ^20^, ^21^, ^22^]

RAFT‐HDA relies on RAFT agents possessing a C=S double bond with a specifically adjusted electron deficiency. The latter should be sufficiently high to enable HDA with a range of dienes, yet not too high in order for a well‐controlled RAFT polymerization to take place. While RAFT‐HDA in organic solvents requires heat, catalysts, or highly active diene partners (e.g., cyclopentadiene or *o*‐quinodimethanes),[^18^, ^19^, ^20^, ^21^, ^22^] we have previously demonstrated that a fast RAFT‐HDA is achieved in aqueous solutions simply by mixing the components at ambient temperature and in the absence of a catalyst, even with less reactive dienes.^[23]^ Such mild conditions seem ideal for the functionalization of proteins, which are generally sensitive to heat or additives. Importantly, most if not all cycloadditions are biorthogonal, thus offer an ideal platform to specifically conjugate polymers to biomolecules.^[24]^ In the present contribution, we report the first protein‐polymer conjugates obtained through the RAFT‐HDA pathway (Scheme 1).

**Scheme 1:**
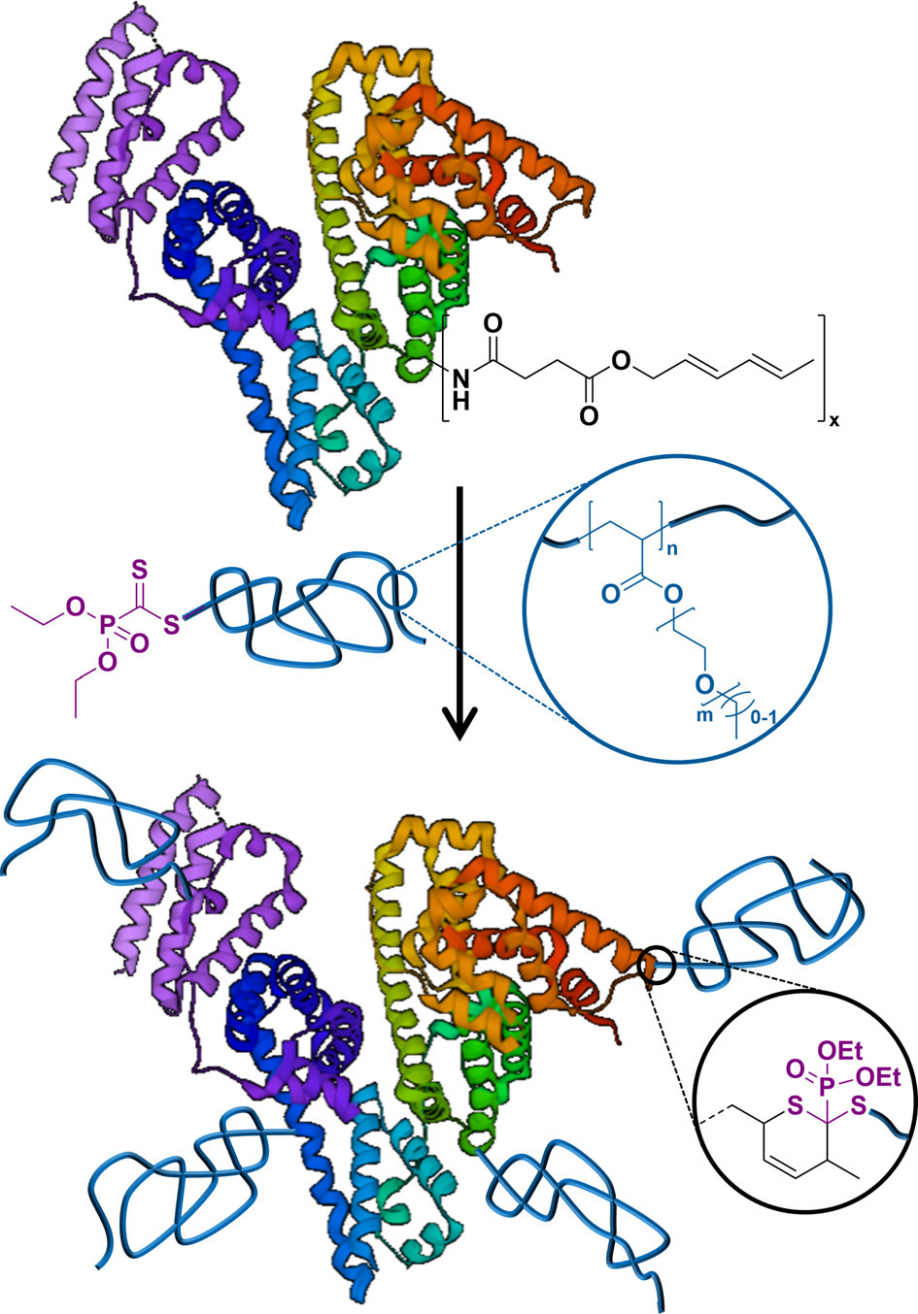
Synthesis of bovin serum albumin polymer conjugates by RAFT‐HDA, as described in the current contribution.

A range of water‐soluble acrylic polymers based on ethylene glycol side chains were first synthesized: (i) homopolymers of triethylene glycol methyl ether acrylate (PmTEGA) and (ii) copolymers of diethylene glycol ethyl ether acrylate and oligoethylene glycol methyl ether acrylate (P(eDEGA‐*co*‐mOEGA)). PmTEGA is water‐soluble in the useful temperature range of PPCs (<55–70 °C)[^25^, ^26^, ^27^, ^28^] and may impart a stealth character and improved solubility to proteins. P(eDEGA‐*co*‐mOEGA)s exhibit a lower critical solution temperature (LCST), which depends on the exact comonomer ratio,[^29^, ^30^] and will lead to thermoresponsive PPCs at possibly useful temperatures. Responsive PPCs[^31^, ^32^] are particularly interesting for control of biomolecular activity,^[33]^ triggered reversible self‐assembly into biohybrid nanostructures[^34^, ^35^, ^36^, ^37^, ^38^] and fast removal/recovery of proteins from solution.[^39^, ^40^] Interestingly, similar polymers with short oligoethylene glycol side chains were shown not to exhibit the non‐desired antigenicity of PEG.^[41]^ 2‐cyanoprop‐2‐yl diethoxyphosphoryldithioformate (**CPDPDT**) was employed as CTA because it simultaneously enables the controlled polymerization of acrylates and the synthesis of polymers with a terminal C=S bond sufficiently electron‐deficient for a rapid HDA cycloaddition to occur.^[23]^ As seen in Figure 1, PmTEGAs with number‐average molar masses *M*_n_ of 2000 and 6000 g mol^−1^ and narrow dispersities (*Đ*=1.1–1.2) were obtained (noted as **PmTEGA2000** and **PmTEGA6000**, respectively). Similarly, P(eDEGA‐*co*‐mOEGA)s **CoP15 000** and **CoP18000**, with *M*_n_ of 15000 and 18000 g mol^−1^ (*Đ*=1.3), respectively, were produced by **CPDPDT**‐mediated RAFT copolymerization. All polymers displayed the classic maximum of absorption at 327 nm (Figure S8), characteristic of the π → π* transition in the diethoxyphosphoryldithioformate end group.

**Figure 1:**
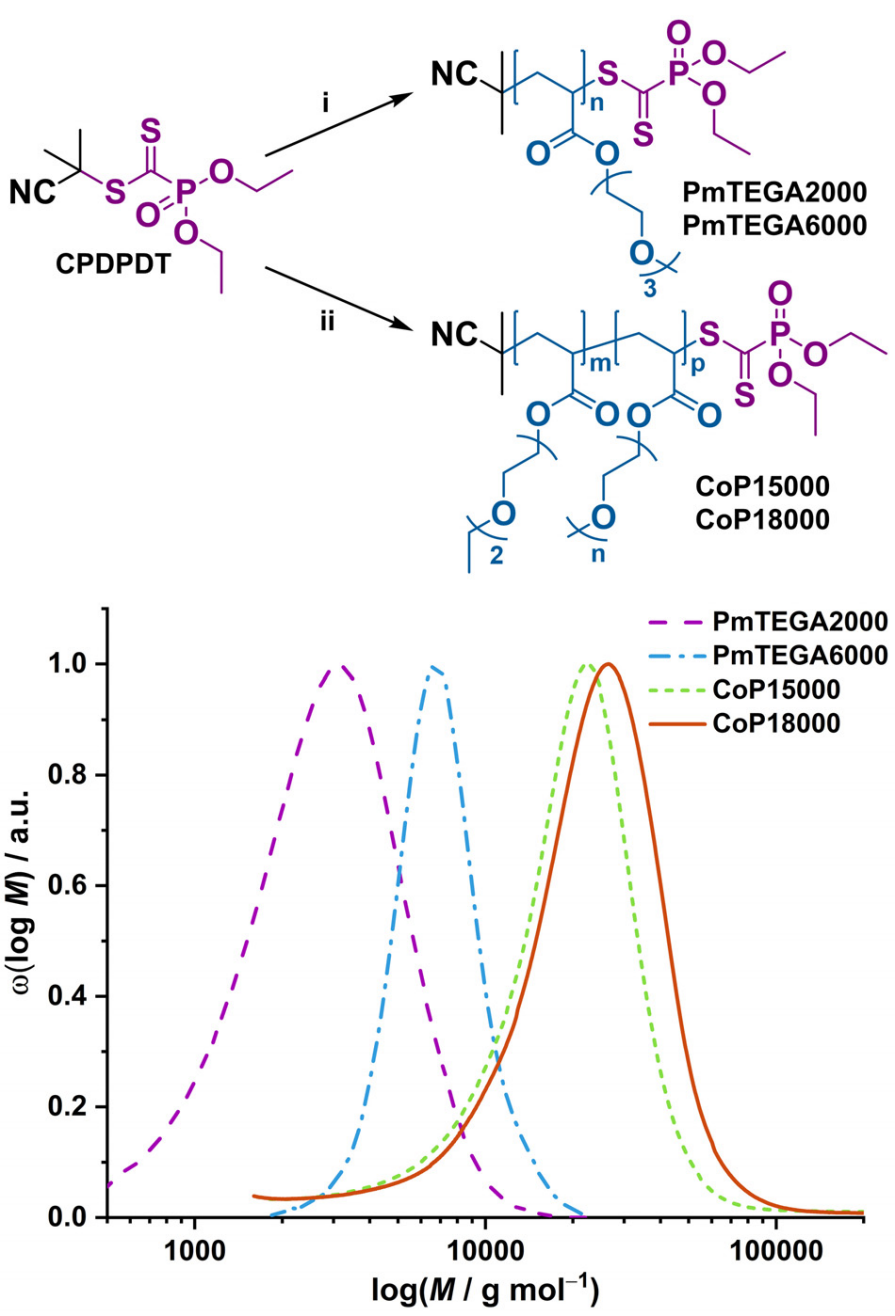
(Top) Synthetic route for oligoethylene glycol‐based polyacrylates by RAFT polymerization in the presence of 2‐cyanoprop‐2‐yl diethoxyphosphoryldithioformate (CPDPDT). (i) mTEGA, AIBN, ethanol, 60 °C. (ii) eDEGA:mOEGA 80/20 mol/mol, AIBN, ethanol, 60 °C. (Bottom) Corresponding SEC traces.

For conjugation reactions not based on natural amino acids, reactive proteins can be obtained either by genetic engineering^[42]^ or simple post‐translational chemical modification.^[43]^ Here, we have chosen the latter for its ease of implementation. To introduce diene moieties, the difunctional linker 2,5‐dioxopyrrolidin‐1‐yl (hexa‐2,4‐dien‐1‐yl)succinate **DSS** (see Supporting Information), consisting of a sorbyl group and a succinimidyl ester on either side, was readily synthesized in two steps and reacted with the lysine residues of the model protein bovin serum albumin (BSA). The diene‐functionalized BSA (**dBSA**) remained fully soluble in aqueous medium and did not show any significant change in circular dichroism (CD) (Figure S14). Mass spectrometry analysis showed the incorporation of an average of 6–7 diene tags per protein molecule, that is, *x=*6–7 in Scheme 1 (Figure S10).

Protein‐polymer conjugation was performed by simple incubation of **dBSA** with the RAFT polymers in aqueous buffers, in the absence of catalyst and at room temperature. During initial experiments, it was observed that the characteristic purple color of the RAFT polymer stock solutions in aqueous media faded with time. It is known that RAFT end groups are sensitive to a range of reagents, mostly primary amines and basic conditions^[44]^ and that discoloration of RAFT polymers implies end‐group loss. For this reason, we monitored this phenomenon by UV/Vis spectroscopic measurements of **PmTEGA6000** solutions in various aqueous buffers. As expected, basic conditions led to the fastest decrease in absorption at 327 nm and consequently the fastest deactivation (Figure S11). Particularly, incubation in bicarbonate buffer at pH 9.15 or Tris buffer at pH 8.1 led to instantaneous full degradation. Phosphate buffer at the same pH was less damaging. Decreasing pH further led to significantly slower degradation. Eventually, sodium phosphate buffer (50 mM at pH 6.0) was chosen for bioconjugation as it offered the best compromise between the slow degradation and close‐to‐neutral conditions. Before protein‐polymer conjugation was addressed, model HDA conjugation reactions with **DSS** were performed. It was observed that absorption at 327 nm decreased significantly faster in reaction mixtures of **PmTEGA6000** and **DSS** compared to the polymer alone (Figure S12). Higher amounts of **DSS** (2 and 3 equivalents) led to faster disappearance of the characteristic RAFT moiety absorption, a clear sign of the HDA reaction occurring at the C=S double bond.^[23]^

A range of conditions were assessed in order to determine the optimum conditions for polymer bioconjugation. Mixtures with various **PmTEGA6000**:**dBSA** molar ratios were prepared in sodium phosphate buffer at pH 6.0 for overnight reactions. As observed in gels obtained by SDS‐PAGE and the corresponding intensity plots (Figure 2 A), higher polymer:protein ratios generated species with higher molar masses. With 80 equiv. and above, conjugates with an average of 6 to 7 grafted polymer chains were obtained, as deduced from the ≈40 kDa shift. It can thus be assumed that in these conditions all accessible diene groups within the protein have been coupled via HDA reaction. Moreover, the kinetics of the reaction was monitored using a 100‐fold molar excess of polymer (Figure 2 B). The reaction was arrested at various incubation times by removing the non‐reacted **PmTEGA6000** by size‐exclusion centrifugation. We observed that the conjugation of the first two polymer chains to **dBSA** occurred within just 1–2 h, with the fully conjugated protein obtained after 6 h. The first easily reachable diene moities might react fast, while the attachment of further polymer chains is certainly slowed down for more buried dienes and due to the increasing steric constraints imposed by already grafted polymer chains. Moreover, the successful preparation of **dBSA‐PmTEGA2000** and **dBSA‐PmTEGA6000** conjugates was also confirmed by a shift in the hydrodynamic diameter distribution compared to that of the free protein, as measured by dynamic light scattering (DLS) (Figure S13). Again, the secondary structure of the protein was not affected by the modification, as demonstrated by CD (Figure S14). Albeit not an enzyme, BSA possesses an esterase activity that can be exploited to further assess modifications. In a colorimetric glyceryl acetate‐based esterase assay (Figure S16), no significant difference between BSA and **dBSA‐PmTEGA6000** were observed, which confirms the conservation of the protein structure and stability.

**Figure 2:**
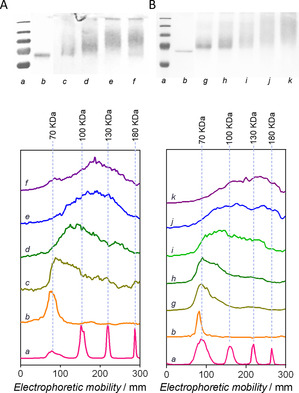
Coomassie‐stained SDS‐PAGE gels of BSA conjugates obtained by RAFT‐HDA with **PmTEGA6000** (up) and corresponding electrophoretograms (bottom). (A) Variation of the [**PmTEGA6000**]:[**dBSA**] ratio for a fixed reaction time of 12 h: 25 (*c*), 50 (*d*), 80 (*e*), and 100 (*f*). (B) Variation of the reaction time for a fixed [**PmTEGA6000**]:[**dBSA**] ratio of 100: 1 h (*g*), 2 h (*h*), 4 h (*i*), 6 h (*j*), and 8 h (*k*). Molecular weight protein ladder (*a*) and control sample (*b*, **dBSA**) are added as references.

The conjugation of functional polymers to proteins leads to PPCs with specific properties. Here, the RAFT‐HDA conjugation was carried out with the thermoresponsive P(eDEGA‐*co*‐mOEGA) copolymers **CoP15000** and **CoP18000** (see Figure 1). DLS measurements revealed a significant increase of the average hydrodynamic diameter from 6.5±0.8 nm for **dBSA** to 7.9±1.2 and 9.1±1.5 nm after the conjugation reaction with **CoP15000** and **CoP18000**, respectively (Figure 3), confirming successful conjugation. As for **PmTEGA6000**, CD measurements revealed no alteration in the secondary structure of BSA through the grafting of **CoP15000** and **CoP18000** (Figure S15).

**Figure 3:**
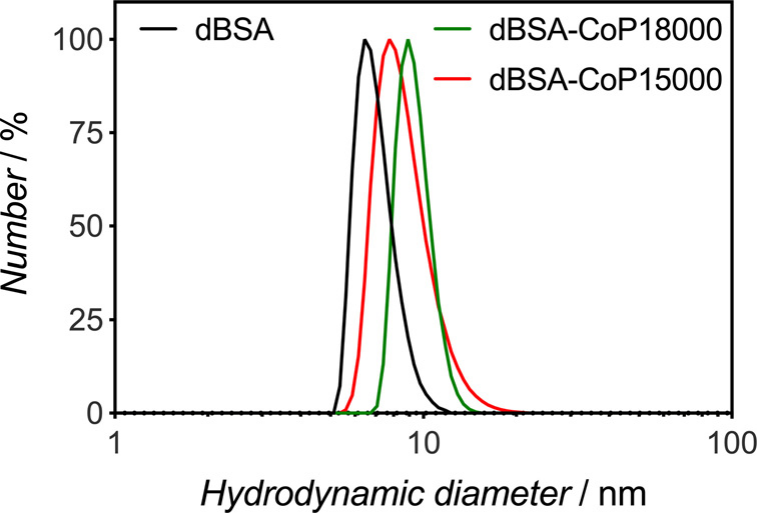
Number‐based hydrodynamic diameter distributions for BSA conjugates obtained by RAFT‐HDA with P(eDEGA‐*co*‐mOEGA) copolymers **CoP15000** and **CoP18000. dBSA** is shown as reference.

The thermoresponsive behavior of the newly generated BSA conjugates was subsequently evaluated. P(eDEGA‐*co*‐mOEGA) copolymers typically exhibit a tunable thermoresponsive behavior over the 25–75 °C range, depending on their comonomer composition and molar mass.[^29^, ^30^] The thermal transition is readily detectable via an increase in the turbidity of the sample, which can be measured within a UV/Vis spectrophotometer (Figure 4 A). We applied a heating ramp from 40 to 55 °C to aqueous solutions of **CoP15000** and **CoP18000** polymers and monitored the absorbance at 670 nm, a wavelength at which no interference with possible chromophores may occur. We observed that while **CoP18000** showed turbidity at temperatures higher than 45 °C, the turbidity of **CoP15000** sample was detected only above 48 °C. We measured cloud points (temperature at 50 % of the maximal absorbance) of 49.5 and 46.5 °C for **CoP15000** and **CoP18000** polymers, respectively.

**Figure 4:**
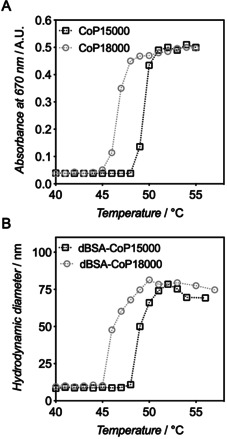
(A) Evolution of the turbidity measured at 670 nm with temperature for aqueous solutions of **CoP15000** and **CoP18000** (15 μM in PBS). (B) Evolution of number‐average hydrodynamic diameters of **dBSA**‐**CoP15000** and **dBSA**‐**CoP18000** conjugates with temperature as measured by DLS. In both cases, measurements were carried out with 1 °C increments and a stabilitization time of 5 min before acquisition.

Finally, we assessed how the thermoresponsive properties of P(eDEGA‐*co*‐mOEGA) copolymers transferred to their corresponding BSA conjugates. Note that BSA was previously shown to be stable in the considered temperature range (i.e., below 60 °C)[^45^, ^46^] and that one could in any case modulate the transition temperatures by varying the compositions of the copolymers. As the turbidity of the medium implied the temperature‐induced aggregation of the polymeric components, the overall size of the PPCs is expected to increase at temperatures higher than LCST, with the protein component stabilizing the aggregates.^[34]^ Purified PPCs were thus subjected to a heating ramp and the hydrodynamic diameter of the particles was simultaneously measured. As shown in Figure 4 B, the average hydrodynamic diameter increased from 7.9±1.2 and 9.1±1.5 nm at 40 °C to 69±5 and 77±4 nm at 55 °C for **dBSA‐CoP15000** and **dBSA‐CoP18000** conjugates, respectively. Interestingly, the onsets of aggregation of the PPCs match well those measured for the polymers alone, and the cloud points only slightly decrease: 48.6 and 45.8 °C for **dBSA‐CoP15000** and **dBSA‐CoP18000**, respectively, versus 49.5 and 46.5 °C for their corresponding free polymers. The polymers are physically bound to the protein counterpart and the protein does not seem to significantly interfere in the temperature‐induced physical aggregation of the polymers.

In conclusion, we introduce the application of the RAFT‐HDA chemistry for catalyst‐free protein‐polymer conjugation under mild conditions (ambient temperature, near‐neutral pH). Using this method, we have decorated the surface of BSA with up to 7 hydrophilic polymer chains, as well as with thermoresponsive polymers. The reaction of the diene‐functionalized protein with the RAFT‐derived diethoxyphosphoryldithioester polymer is relatively fast, pH‐dependent, and can be monitored by UV/Vis spectroscopy and SDS‐PAGE. Finally, we demonstrate that the thermoresponsive properties of the polymers are transferred to the protein‐polymer conjugates, showing similar phase‐separation temperatures. The current procedure entails pre‐conditioning of the protein through covalent anchoring of reactive diene tags. Further control of the bioconjugation degree and location is certainly achievable by the introduction of genetically encoded unnatural aminoacid containing the diene group.[^42^, ^47^]

## Conflict of interest

The authors declare no conflict of interest.

## Supporting information

As a service to our authors and readers, this journal provides supporting information supplied by the authors. Such materials are peer reviewed and may be re‐organized for online delivery, but are not copy‐edited or typeset. Technical support issues arising from supporting information (other than missing files) should be addressed to the authors.

Supplementary
