# Supplementary material for: Hetero‐Diels–Alder Cycloaddition with RAFT Polymers as Bioconjugation Platform
Source: Angew Chem Int Ed Engl. 2020 Sep 2;59(45):19951–5. doi: 10.1002/anie.202005747 (PMC7693046; doi:10.1002/anie.202005747)
Supplement: Supplementary file 1 — Supplementary [file ANIE-59-19951-s001.pdf]

Angewandte Chemie
Eine Zeitschrift der Gesellschaft Deutscher Chemiker

Supporting Information

Hetero-Diels–Alder Cycloaddition with RAFT Polymers as Bioconjugation Platform

Ana Beloqui, Shivshankar R. Mane, Marcel Langer, Mathias Glassner, Dennis M. Bauer, Ljiljana Fruk, Christopher Barner-Kowollik, and Guillaume Delaittre**

anie_202005747_sm_miscellaneous_information.pdf

Table of Contents

Materials **S1**
Characterization **S1**
Synthesis **S2**
*4-((Hexa-2,4-dien-1-yl)oxy)-4-oxobutanoic acid (***1***)* S2
*2,5-Dioxopyrrolidin-1-yl (hexa-2,4-dien-1-yl)succinate (***DSS***)* S3
Triethylene glycol methyl ether acrylate (mTEGA) S5
*RAFT polymerization of mTEGA (***PmTEGA2000***)* S6
RAFT copolymerization of eDGA and mOEGA S8
Introduction of diene moieties in BSA **S9**
RAFT end group stability in aqueous media **S9**
RAFT-HDA test reaction **S10**
Protein–polymer bioconjugation **S11**
References **S13**
Author Contributions **S13**

Materials

Acryloyl chloride (abcr, 96%), bovine serum albumin (BSA; Sigma, IgG-free), *N,N*-diisopropylethylamine (DIPEA; TCI, ≥ 99%), 1-ethyl-3-(3-dimethylaminopropyl) carbodiimide hydrochloride (EDC.HCl; Carl Roth, ≥ 99 %), *N*-hydroxysuccinimide (NHS; Acros, 98+%), succinic anhydride (Alfa Aesar, 99%), *trans*,*trans*-2,4-hexadien-1-ol (Sigma, 97%), triethylene glycol monomethyl ether (Sigma, ≥ 97.0%), triethylamine (TEA; Fisher, > 95%), glyceryl triacetate (Sigma, 99%), phenol red (Fisher, 99%), citric acid (Roth, ≥ 99.5%), hydrochloric acid (HCl; Roth, 34%), magnesium sulfate hydrate (Roth, > 99%), potassium chloride (Acros, 99+%), sodium acetate trihydrate (Roth, ≥ 99.5%), sodium bicarbonate ($NaHCO_3$; Roth, ≥ 99%), sodium citrate monobasic anhydrous (Sigma, ≥ 99.5%), sodium chloride (NaCl; Roth, ≥ 99.8%), sodium phosphate dibasic (Sigma, ≥ 99%), phosphate buffered saline (PBS; Sigma), sodium sulfate (Roth, ≥ 99%), tris(hydroxymethyl)amino methane (Tris; Roth, ≥ 99%), dichloromethane (DCM; VWR, reagent grade), diethyl ether (Roth, ≥ 99.5%), dimethylsulfoxide (DMSO; Fisher, > 95%), ethanol (Acros, 99.8%), and *n*-hexane (Fisher, ExtraPure) were used as received.
Azobisisobutyronitrile (AIBN; Sigma-Aldrich, 98 %) was recrystallized from ethanol. Di(ethylene glycol) ethyl ether acrylate (eDEGA; Sigma, ≥ 90%) and oligo(ethylene glycol) methyl ether acrylate (mOEGA; M_n = 480 g mol^{-1}; Sigma) were eluted through a basic alumina column (Roth) to remove the inhibitor.
The synthesis of 2-cyanoprop-2-yl diethoxyphosphoryldithioformate (**CPDPDT**)[1] and **PmTEGA6000** (M_n = 6000 g mol^{-1}; $Đ$ = 1.13)[2] were previously reported.

Characterization

^{1}H nuclear magnetic resonance (NMR) spectroscopy was performed on a Bruker NMR 500 spectrometer at 500 MHz. The samples were dissolved in deuterated solvent. The solvent signals were employed for chemical shift corrections.

Size-exclusion chromatography was carried out a TOSOH Eco-SEC HLC-8320 GPC System comprising an autosampler, a SDV 5 µm bead size guard column (50 × 8 mm, PSS) followed by three SDV 5 µm columns (300 × 7.5 mm, subsequently 100 Å, 1000 Å, and 10^5 Å pore size, PSS), and a differential refractive index (DRI) detector, using tetrahydrofuran (THF) as the eluent at 30 °C with a flow rate of 1 mL min^{-1}. The SEC system was calibrated with linear PMMA standards ranging from 800 to 1.82 × 10^6 g mol^{-1}.

UV-Vis spectroscopy was performed with a Biotek Epoch 2 spectrophotomer in 96-well plates. 200 µL of sample were used for measurements, which were all performed in triplicate. The turbidity measurements were performed by progressively heating the sample (increments of 1 °C), with 5 min allowed for stabilization at each step.

Sodium dodecylsulfate polyacrylamide gel electrophoresis (SDS-PAGE) was used to analyze protein-polymer conjugates on 12% SDS-PAGE under non-reducing conditions, following the Laemmli protocol.[3]

Dynamic light scattering (DLS) measurements were performed at 25 °C at an angle of 173° (backscattering mode) with a Zetasizer Nano S from Malvern using a 4mW He-Ne laser at 633 nm. Analysis of the data was carried out using the Nano DTS v.5.10 software. Protein–polymer conjugates were diluted to 0.1 mg/mL in PBS solution. Experiments were performed with 12 readouts of 3 independent measurements for each sample. The temperature-dependent aggregation experiment was performed by progressively heating the sample (increments of 1 °C), with 5 min allowed for stabilization at each step.

Matrix-assisted laser desorption ionization coupled to time-of-flight (MALDI-ToF) spectrometry. Mass spectra were acquired with a 4800 Proteomics Analyzer (Applied Biosystems, Foster City, CA, USA) in positive ion linear mode and a mass range of 60 000 to 80 000 Da. The laser intensity was set to 4700. The spectra obtained represent the average of laser shots taken by an automatic scheme measuring spectra over the whole spot. Sinapinic acid was used as matrix.

Circular dichroism (CD) measurements. CD spectra were measured using a Jasco J-815CD Spectrometer. Spectra were acquired at 0.2 mg mL^{-1} BSA concentration in water using 0.1 cm pathlength quartz cuvette. All CD spectra were recorded with a bandwidth of 1 nm at 1 nm increments and 10 s average time, over a wavelength range from 190 to 260 nm.

Synthesis

*4-((Hexa-2,4-dien-1-yl)oxy)-4-oxobutanoic acid (***1***)*

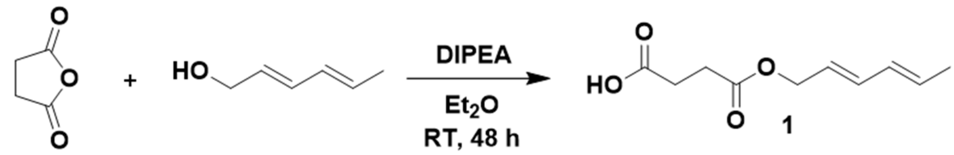

Succinic anhydride (2 g, 19.7 mmol, 1.0 eq.) was dissolved in diethyl ether (10 mL), followed by the addition of *trans*,*trans*-2,4-hexadien-1-ol (2.34 g, 23.1 mmol, 1.2 eq.) and DIPEA (2.63 g, 20.1 mmol, 1.0 eq.). The reaction mixture was then stirred for 48 h at room temperature. The solvent was evaporated. The obtained residue was dissolved in DCM and washed with 5 wt% citric acid (3 × 50 mL). Subsequently, the organic layer was dried over magnesium sulfate and the solvent was evaporated under reduced pressure to yield a brownish solid (yield 92%).

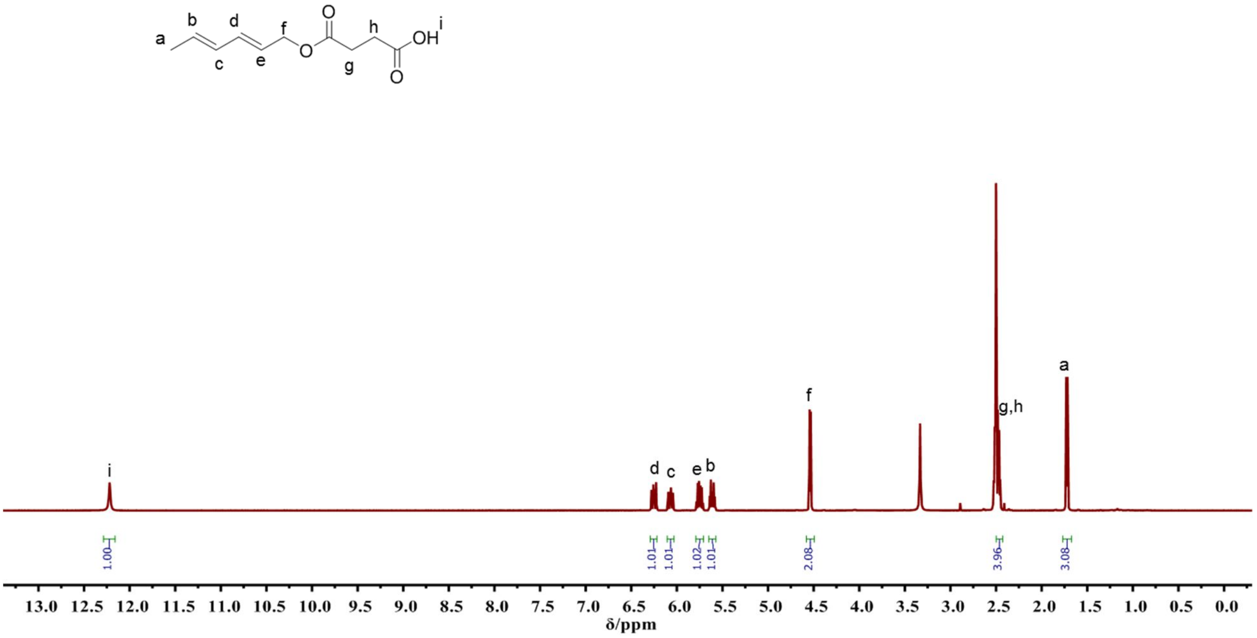

Figure S1. ^1H NMR spectrum of 4-((hexa-2,4-dien-1-yl)oxy)-4-oxobutanoic acid (**1**) in DMSO-*d*6.

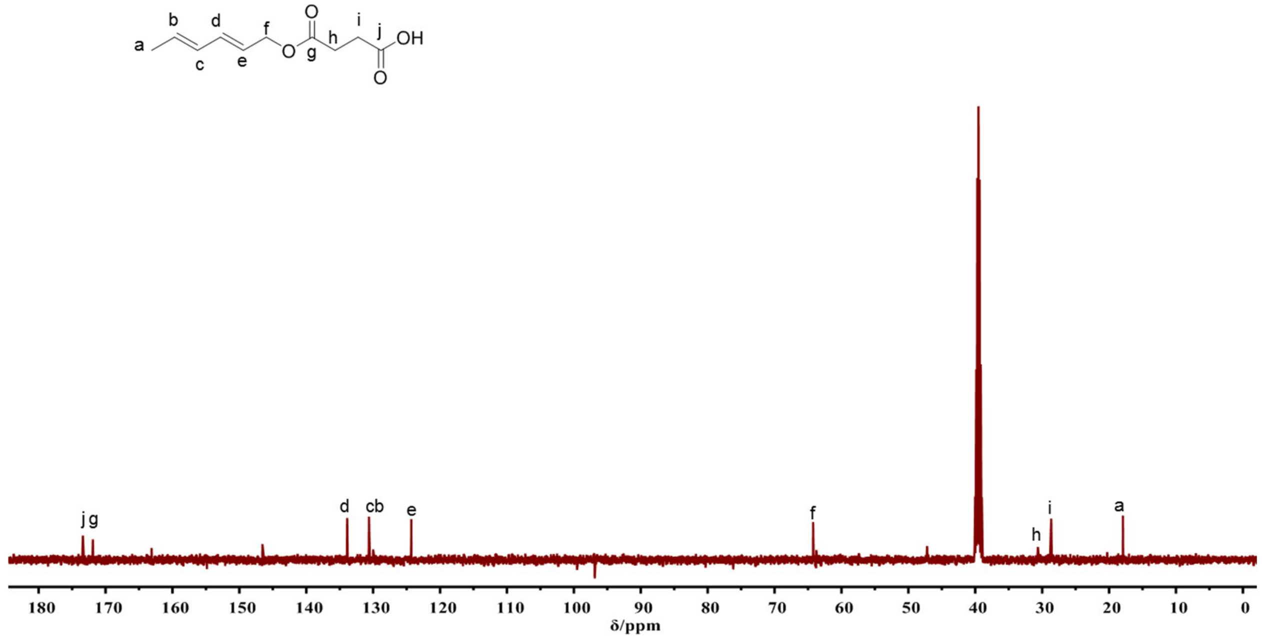

Figure S2. ^{13}C NMR spectrum of 4-((hexa-2,4-dien-1-yl)oxy)-4-oxobutanoic acid (**1**) in DMSO-*d*6.

*2,5-Dioxopyrrolidin-1-yl (hexa-2,4-dien-1-yl)succinate (***DSS***)*

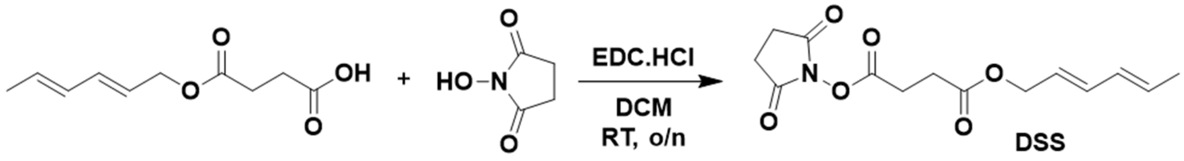

4-((hexa-2,4-dien-1-yl)oxy)-4-oxobutanoic acid **1** (0.500 g, 2.52 mmol, 1.0 eq.) was dissolved in DCM (2.5 mL). EDC.HCl (0.627 g, 3.24 mmol, 1.3 eq.) was added and stirred for 15 min, followed by the addition of NHS (0.580 g, 50.5 mmol, 2.0 eq.). The resulting solution was stirred overnight at ambient temperature. The solvent was evaporated. The residue was redissolved in DCM and subsequently washed with water (3 × 30 mL) and brine (3 × 30 mL). Finally, the organic layer was dried over sodium sulfate and the solvent was evaporated under reduced pressure to obtain **DSS** as a red color oil (yield 69%).

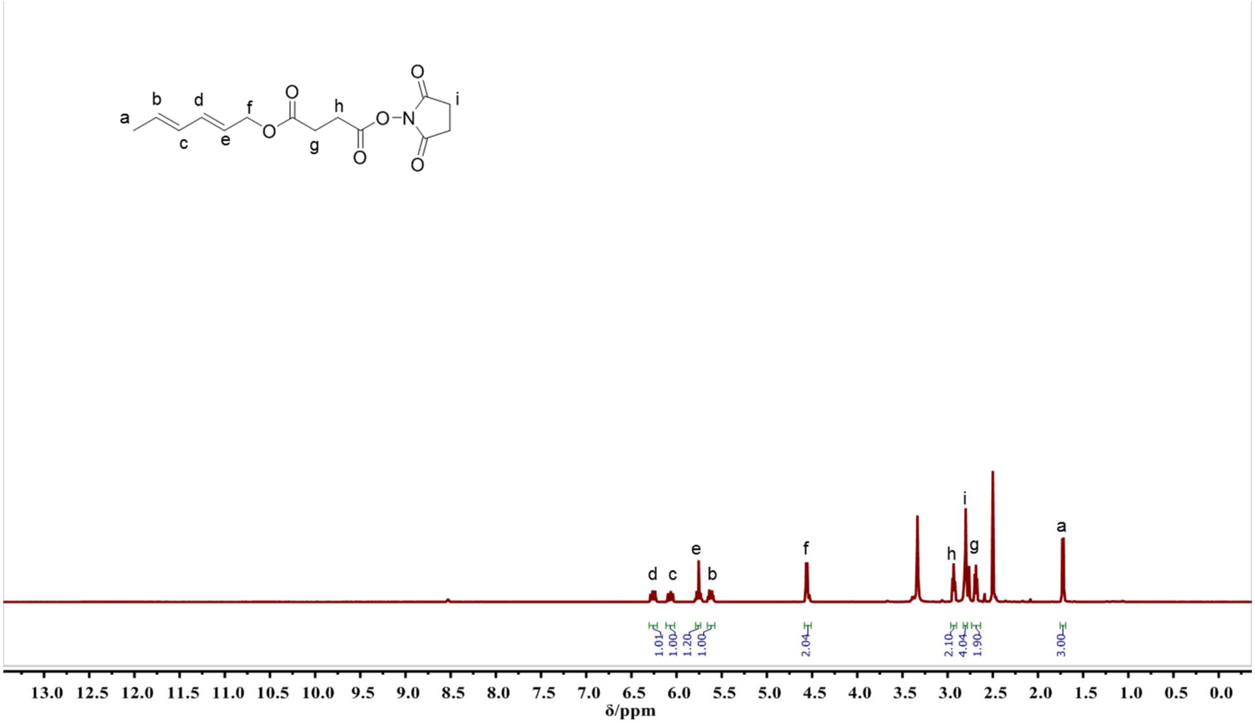

Figure S3. ^{1}H NMR spectrum of DSS in DMSO-*d*6.

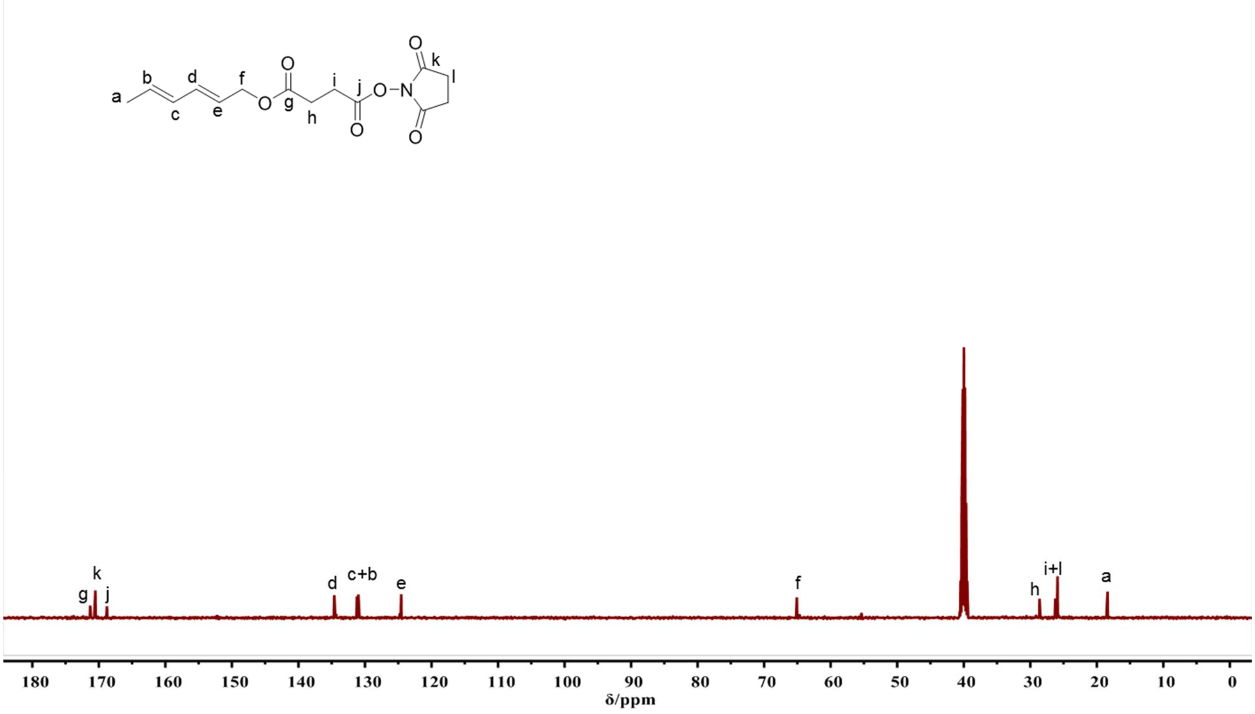

Figure S4. ^{13}C NMR spectrum of **DSS** in DMSO-*d*6.

Triethylene glycol methyl ether acrylate (mTEGA)

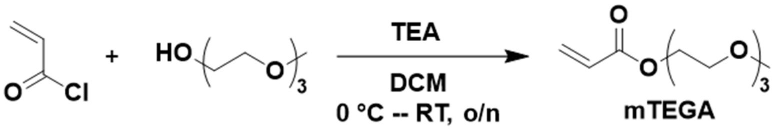

mTEGA was synthesized using a procedure adapted from the literature.[4] Triethylene glycol monomethyl ether (3.0 g, 18.3 mmol, 1.0 eq.) was dissolved in DCM (20 mL). TEA (2.2 g, 20.7 mmol, 1.1 eq.) was added and the reaction flask kept in an ice bath. After 15 min, acryloyl chloride (2.0 g, 21.2 mmol, 1.2 eq.) was added dropwise over 30 min under N_2 atmosphere. After complete addition, the ice bath was removed and the reaction mixture was stirred overnight at RT. The triethylamine salt was filtered off and the solution was diluted with DCM. The product was isolated by sequential washing with aq. $NaHCO_3$, water (3 × 50 mL), and brine (3 × 50 mL). The organic phase was dried over sodium sulfate. The solvent was removed under reduced pressure to yield mTEGA as a colorless oil (yield 66%).

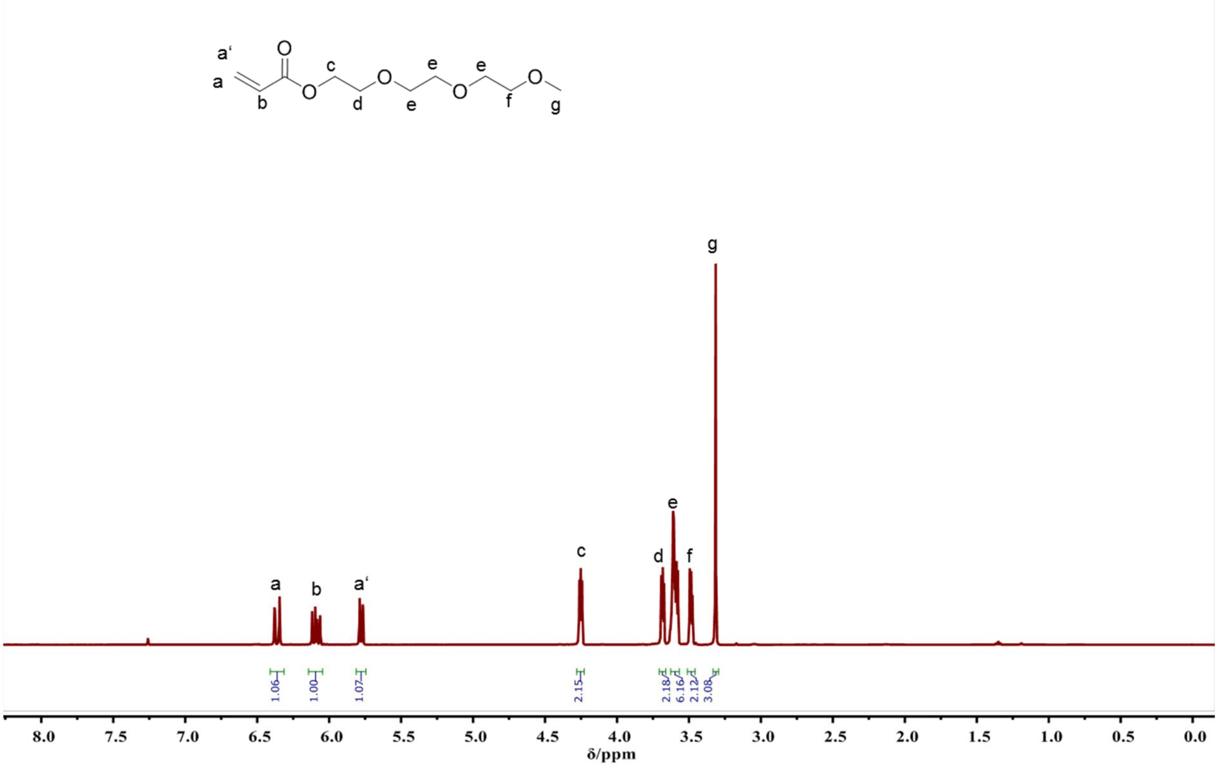

Figure S5. ^{1}H NMR spectrum of mTEGMA in $CDCl_3$.

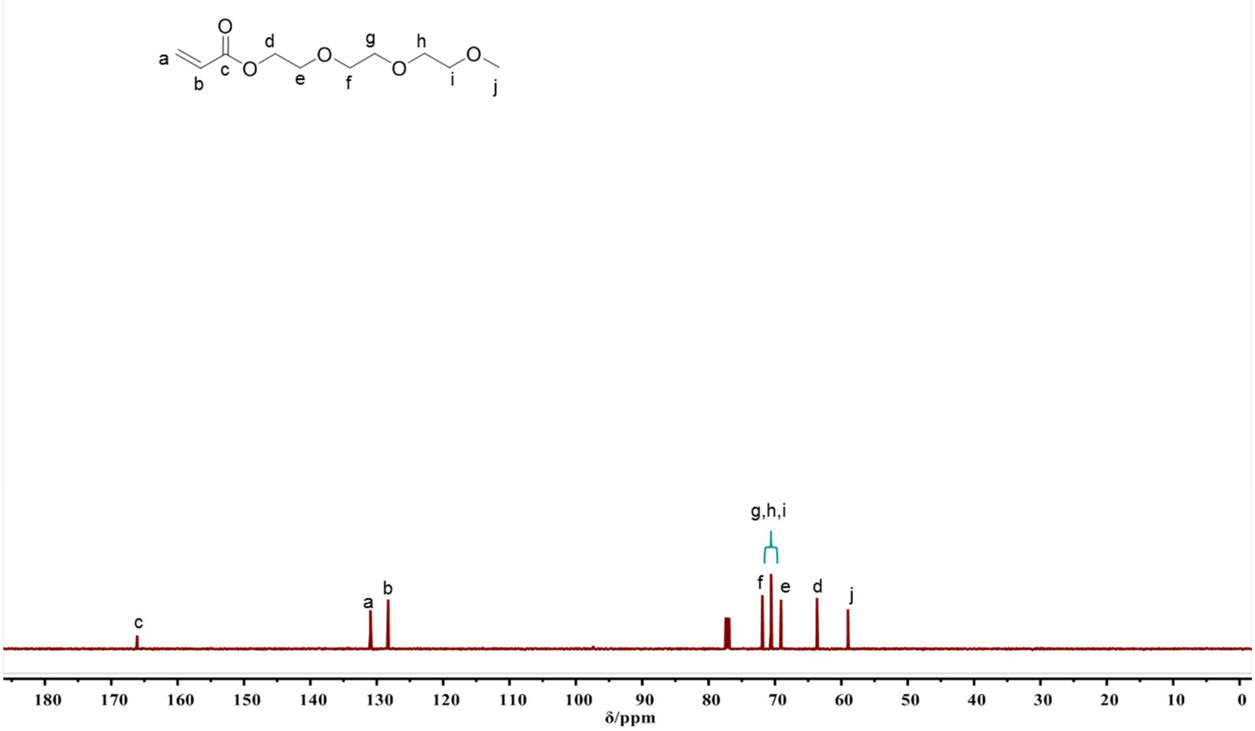

Figure S6. ^{13}C NMR spectrum of mTEGMA in $CDCl_3$.

*RAFT polymerization of mTEGA (***PmTEGA2000***)*

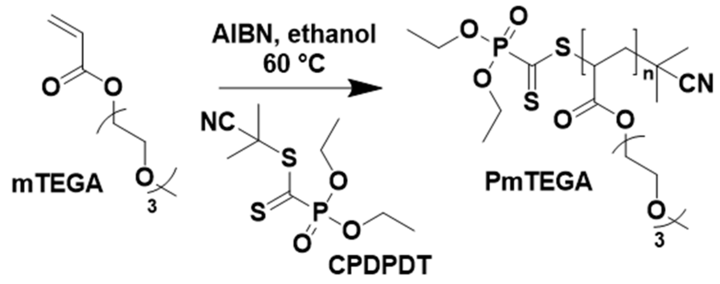

A mixture of CPDPDT (24.3 mg, 0.086 mmol, 1.0 eq.), AIBN (7.1 mg, 0.043 mmol, 0.5 eq.), mTEGA (1 g, 4.6 mmol, 53 eq.), and ethanol (1 g) was placed in a Schlenk flask and deoxygenated by purging with nitrogen for 30 min. Next, the flask was placed in preheated oil bath (60 °C) to commence the polymerization. After 3 h, the flask was taken out and the reaction was stopped via rapid cooling. The polymer was obtained by repeated precipitation in *n*-hexane and decantation.
(SEC) M_n = 2100 g mol^{-1}; $Đ$ = 1.23.

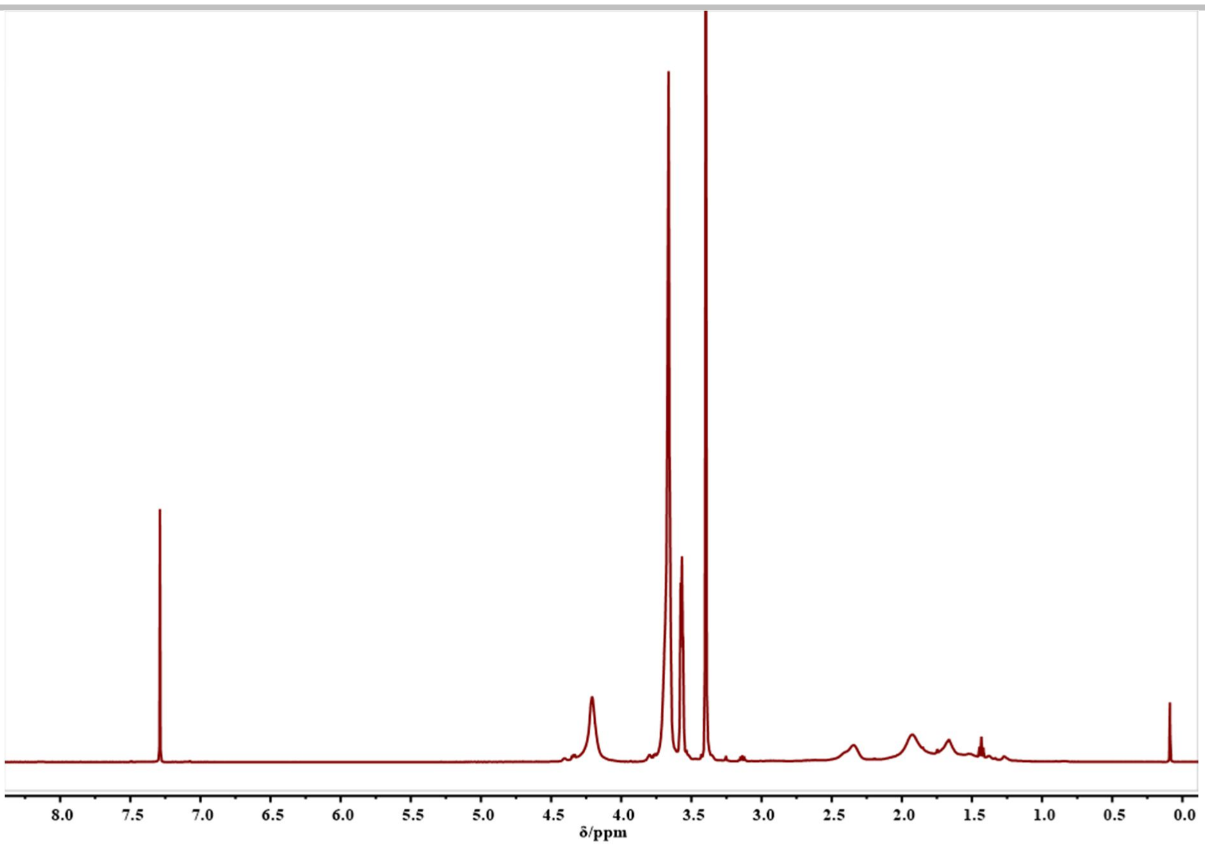

Figure S7. ^1H NMR spectrum of **PmTEGA2000** in $CDCl_3$.

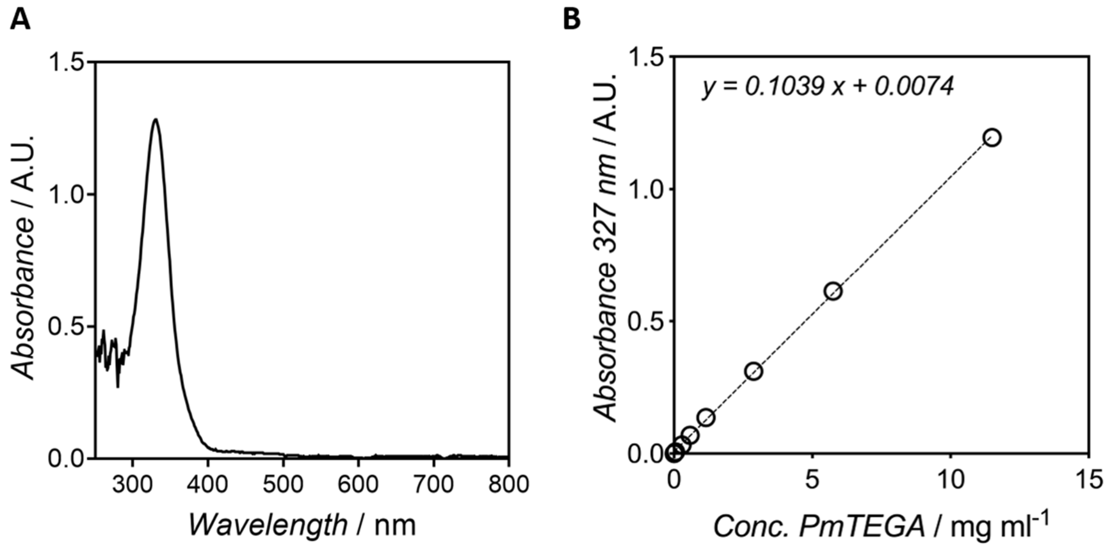

Figure S8. UV–Vis spectrum of **PmTEGA6000** at 10 mg mL^{-1} in water (A) and the correlation of absorbance of **PmTEGA6000** with the concentration of the polymer (B). *Note that measurements were carried out in plastic well-plates, which are not transparent in the UVC region.*

RAFT copolymerization of eDGA and mOEGA

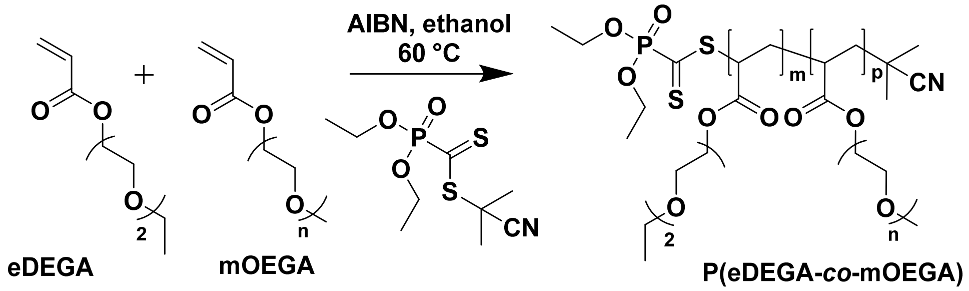

A mixture of CPDPDT (37.3 mg, 0.133 mmol, 1.0 eq.), AIBN (10.8 mg, 0.066 mmol, 0.5 eq.), eDEGA (2 g, 9.6 mmol, 72 eq.), mOEGA (1.276 g, 2.7 mmol, 20 eq.), and ethanol (3.3 g, 4.2 mL) was split between two Schlenk flasks and deoxygenated by purging with nitrogen for 1 hour. The flasks were then immersed in pre-heated oil bath (60 °C) to start the polymerization. The first flask (**CoP15000**) was removed after 4 hours and rapidly cooled down. The same was done with the second flask (**CoP18000**) after 5 hours. The copolymers were obtained by repeated precipitation in cold diethyl ether and drying under vacuum.
(SEC) **CoP15000**: M_n = 14900 g mol^{-1}; $Đ$ = 1.28.
CoP18000: M_n = 17900 g mol^{-1}; $Đ$ = 1.31.

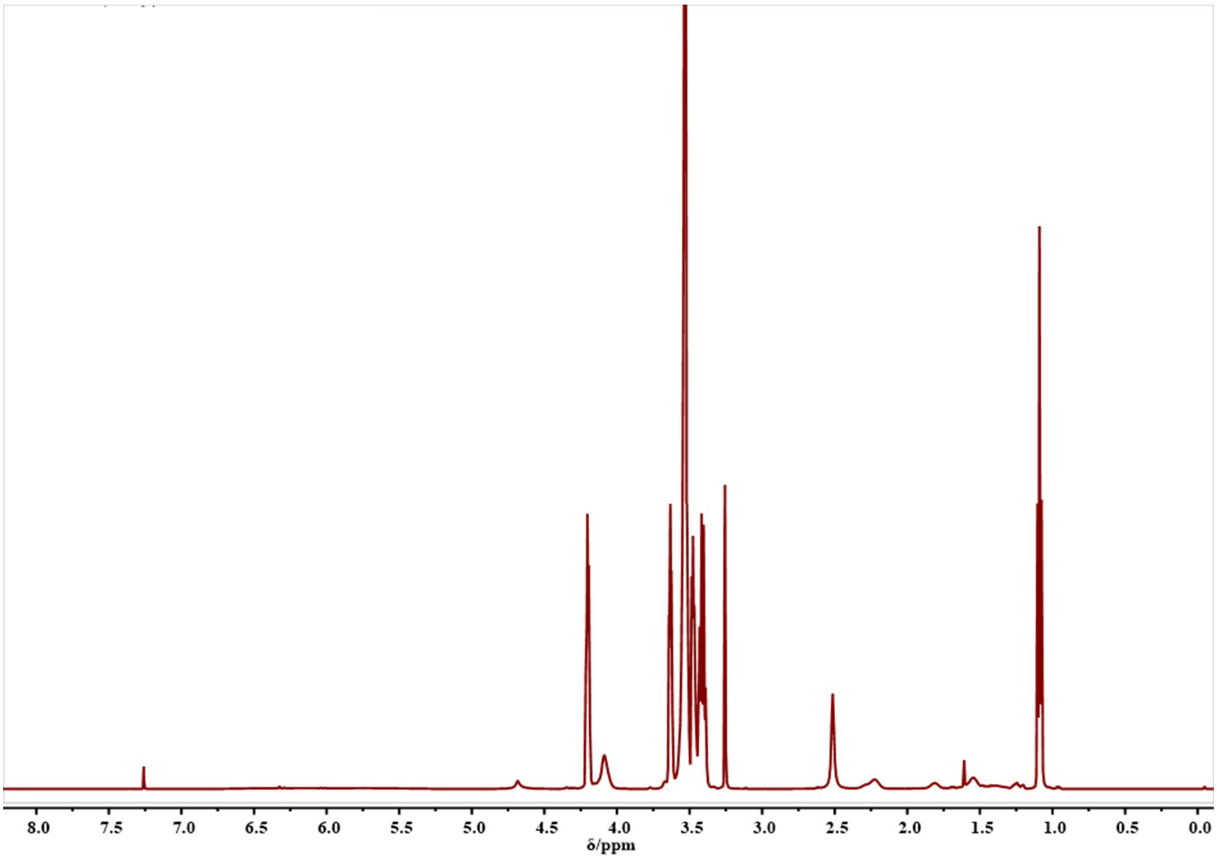

Figure S9. ^{1}H NMR spectrum of P(eDEGA-*co*-mOEGA) **CoP15000** in $CDCl_3$.

Introduction of diene moieties in BSA

Bovine serum albumin (BSA) was taken as model protein to demonstrate the straightforward RAFT-HDA polymer–protein bioconjugation. As a first step, soluble diene-functionalized BSA, **dBSA**, was synthesized by direct coupling of **DSS** (27.8 µmol dissolved in 500 µL of DMSO) to the primary amino groups of a BSA (0.57 µmol in 5 mL of 30 mM sodium phosphate solution at pH 8.4). The reaction mixture was kept at 37 °C for 1 h. Thereafter, unreacted material was removed by filtering the reaction mixture through a 30 kDa MWCO membrane. The modified protein was further washed in sequential filtration steps with a total of 50 volumes of MilliQ water (five steps with 10 volumes of water).
The coupling reaction was confirmed by MALDI-MS (Figure S10), with an increase of m/z value of approx. 1200, which corresponds to the introduction of an average of approx. 6–7 diene units per BSA molecule. The **dBSA** solution was stored at –20 °C until its use.

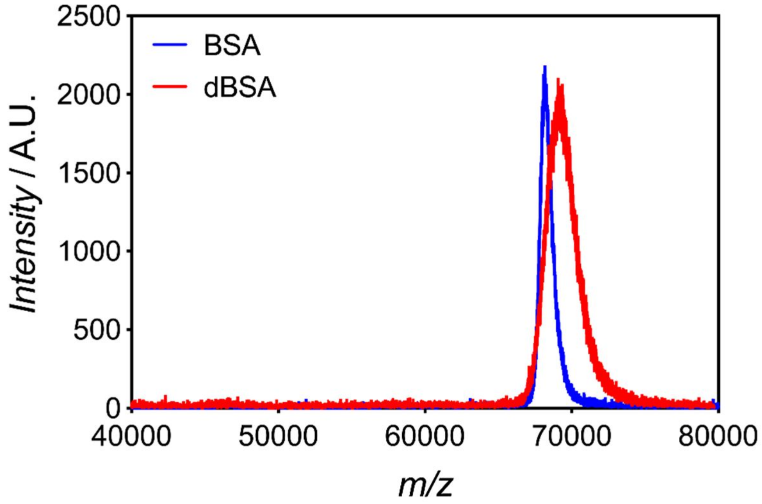

Figure S10. MALDI-ToF spectra of commercial BSA (blue) and its DSS-modified counterpart **dBSA** (red).

RAFT end group stability in aqueous media

The stability of the RAFT end-group of PmTEGA2000 in water and in a range of buffers and pH values was checked by UV-Vis spectroscopy. PmTEGA2000 (0.25 mM) was dissolved in water (pH 6.8), PBS (10 mM sodium phosphate, 135 mM NaCl, 2.5 mM KCl, pH 7.4), sodium citrate (50 mM, pH 4.5), sodium acetate (50 mM, pH 5.6), sodium phosphate (50 mM, pH 6.0 and pH 8.1), Tris-HCl (50 mM, pH 8.1), and sodium bicarbonate (50 mM, pH 9.1). The absorbance of these solutions were immediately monitored at 327 nm for 180 min and plotted as % of the absorbance at t = 0. Experiments were performed in triplicate.

As observed in the Figure S12, the RAFT group is hydrolyzed faster at higher pH values. Moreover, Tris buffer seems detrimental for the stability of RAFT group compared to phosphate buffer at the same pH of 8.1. This effect might be explained by the presence of a primary amine group in Tris and the known susceptibility of the dithioester end-group towards primary amines.[5]

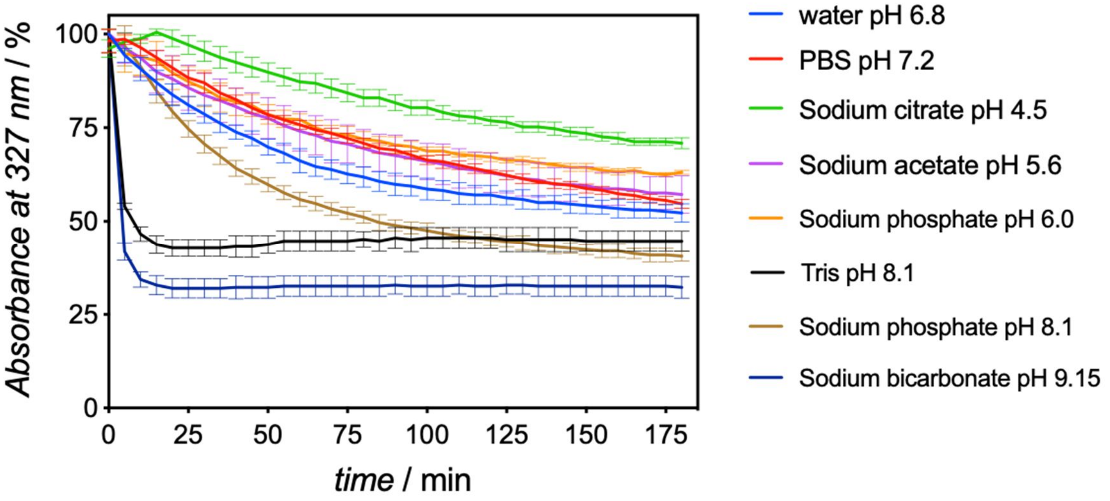

Figure S11. Evolution of the absorbance at 327 nm of aqueous solutions of **PmTEGA6000** in various buffer conditions.

RAFT-HDA test reaction

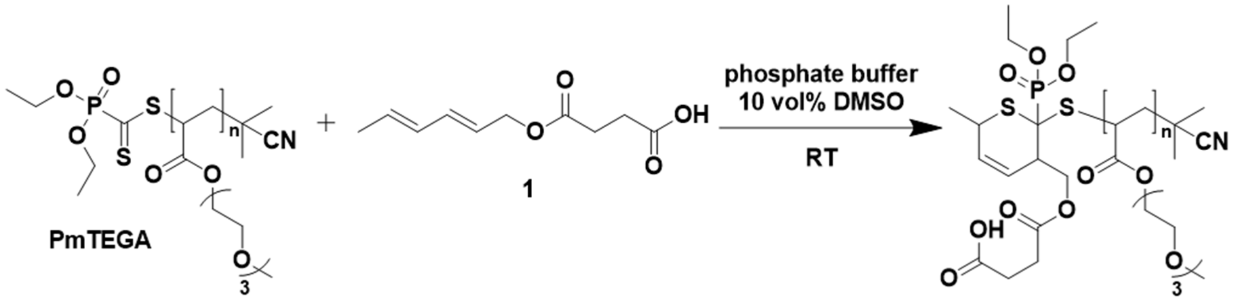

A first HDA test reaction was performed mixing **PmTEGA6000** (0.05 µmol) with 1, 2, and 3 equivalents of **1** in phosphate buffer (50 mM, pH 6.0). DMSO (10%, v/v) was added to solubilize **1**. The absorption of the RAFT group at 327 nm was used to monitor the reaction course. A solution of PmTEGA6000 without diene was used as control.

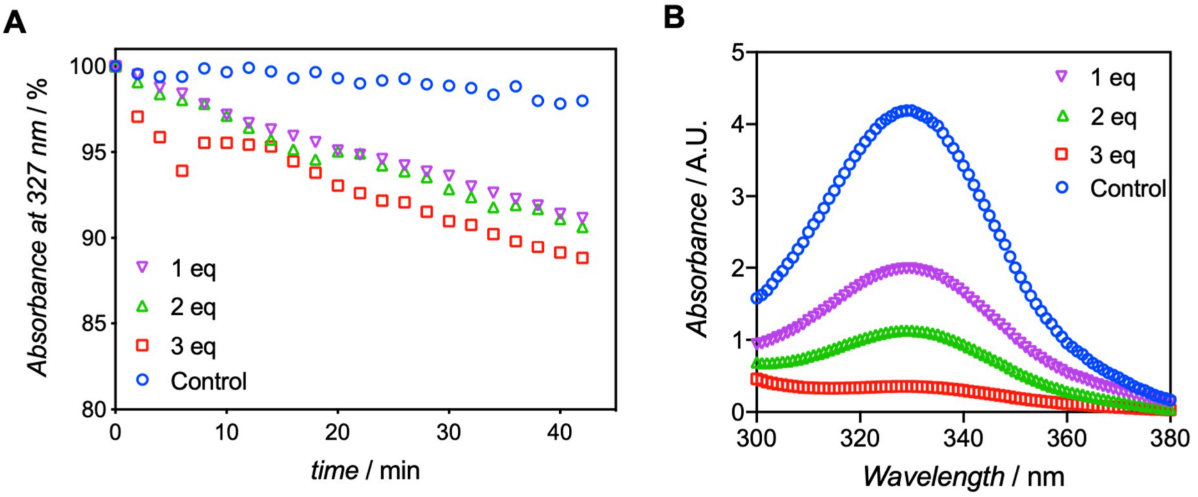

Figure S12. RAFT-HDA test reactions using various equivalents of diene compound **1** with **PmTEGA6000** (40 mg mL^{-1}). A control sample (**PmTEGA6000** without diene) is introduced as reference. A) Short-time kinetics of RAFT-HDA reaction monitored at 327 nm. B) UV-Vis spectra of solutions after overnight reaction.

Protein–polymer bioconjugation

A typical RAFT polymer–protein HDA bioconjugation experiment was performed by mixing **dBSA** (70 µM) with a RAFT polymer (100 equivalents, 7 mM) in sodium phosphate buffer (30 mM, pH 6.0). The reaction was stirred overnight at room temperature. Unreacted material was removed by sequential filtration steps through a 30 KDa cutoff membrane. Protein–polymer conjugates were characterized by DLS and gel electrophoresis (SDS-PAGE).

Additional dynamic light scattering data

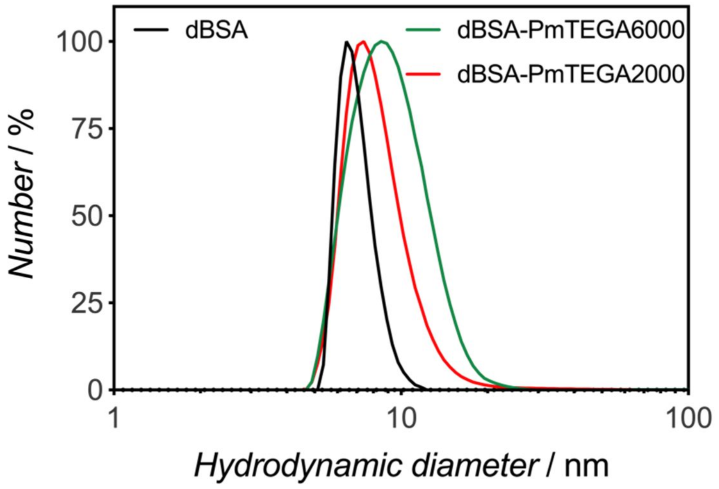

Figure S13. Number-based hydrodynamic diameter distributions obtained by DLS for **dBSA** and its polymer conjugates with PmTEGAs.

Circular dichroism data

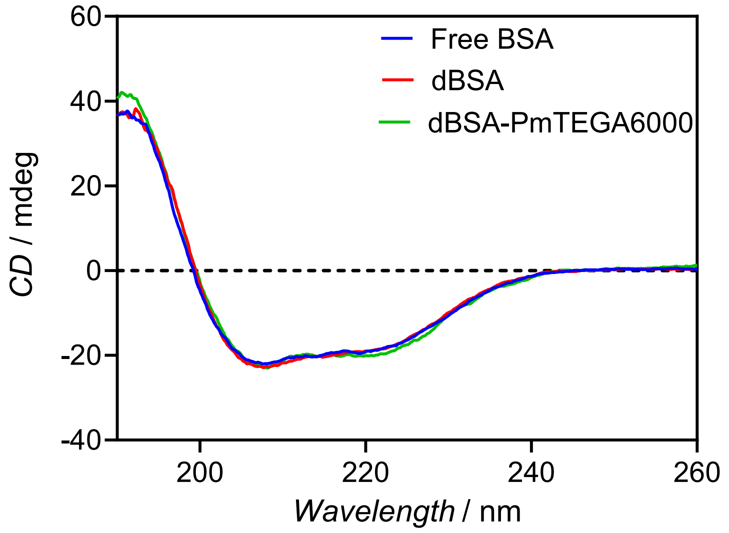

Figure S14. CD spectra of free BSA, **dBSA**, and **dBSA-PmTEGA6000** polymer with an [PmTEGA6000]:[dBSA] molar ratio of 100 (8 h of reaction, sample shown in Figure 2B-*k*). Samples were measured in water at a concentration of 0.2 mg mL^{-1} of BSA.

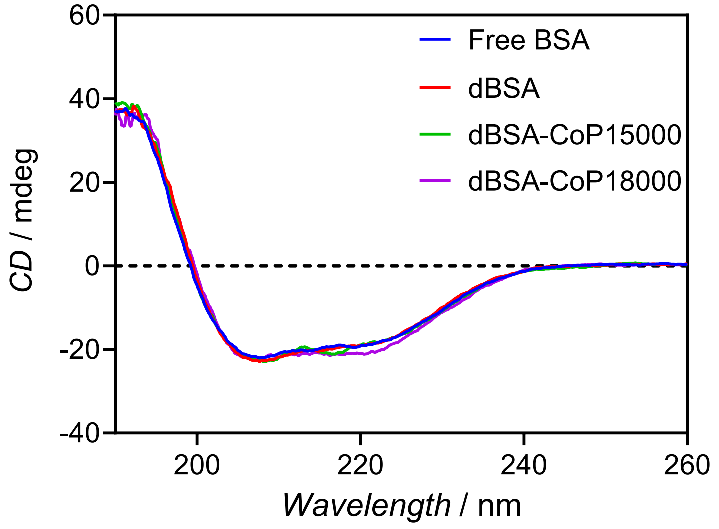

Figure S15. CD spectra of free BSA, **dBSA**, **dBSA-CoP15000**, and **dBSA-CoP18000**. Samples were measured in water at a concentration of 0.2 mg mL^{-1} of BSA.

Esterase assay

Glyceryl triacetate was used as substrate and phenol red as pH indicator. The hydrolytic activity of BSA leads to the release of acetic acid and to the subsequent decrease of pH, which can be monitored by the decrease of the absorbance at 560 nm, resulting from protonation of the phenol red dianion. Reactions were performed using a mixture of phenol red (1 mM), glyceryl acetate (5%, v/v), BSA (either free or conjugated version, 0.2 mg mL^{-1}) in phosphate buffer (30 mM, pH 8.3). The absorbance at 560 nm was recorded by taking aliquots at different reaction times (from 0 to 30 min).

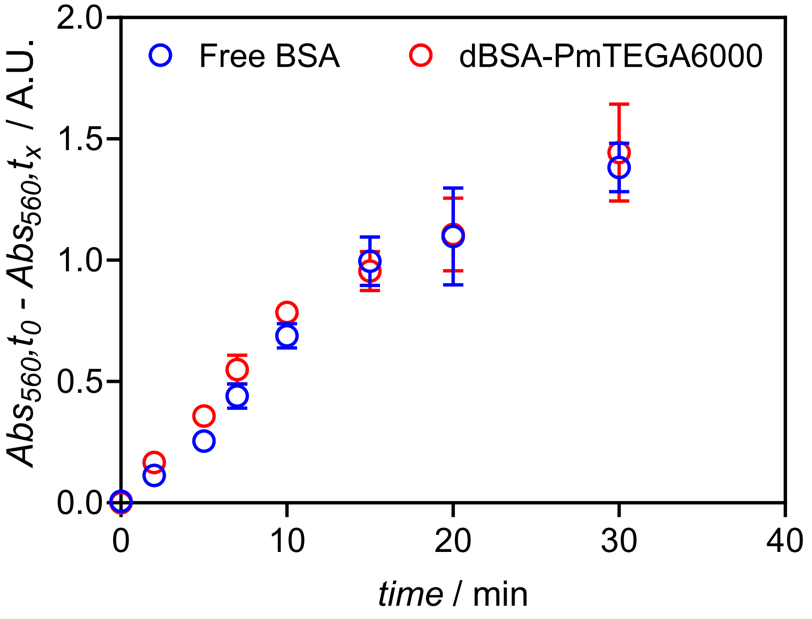

Figure S16. Esterase activity plots of free BSA and conjugated protein dBSA-PmTEGA6000. The decrease of the maximum at 560 nm of the phenol red is monitored at different reaction times.

References

[1] A. Alberti, M. Benaglia, M. Laus, K. Sparnacci, *J. Org. Chem.* **2002**, *67*, 7911–7914.
[2] B. Vonhören, M. Langer, D. Abt, C. Barner-Kowollik, B. J. Ravoo, *Langmuir* **2015**, *31*, 13625–13631.
[3] U. K. Laemmli, *Nature* **1970**, *227*, 680–685.
[4] F. Hua, X. Jiang, D. Li, B. Zhao, *J. Polym. Sci. Part A Polym. Chem.* **2006**, *44*, 2454–2467.
[5] H. Willcock, R. K. O'Reilly, *Polym. Chem.* **2010**, *1*, 149–157.

Author Contributions

A.B. performed organic synthesis, bioconjugation, and characterization of the bioconjugates. S.R.M., M.L., and M.G. performed organic and polymer synthesis. D.M.B. and L.F. contributed to preliminary studies on the RAFT-HDA concept. C.B.-K. conceived the RAFT-HDA concept and acquired funding. G.D. originated and supervised the current bioconjugation study, acquired funding, and wrote the article. All authors reviewed the paper and contributed additional writing.
